# Progress in Genito-Urinary Surgery

**Published:** 1902-09-27

**Authors:** 


### Progress in Genito-Urinary Surgery.

(*Continued from page* 435.)

**Elephantiasis of the External Genital Organs.** J. P. zum Busch [4] has published notes of four cases of the above condition, not due to the filaria sanguinis hominis, but following the removal of the inguinal lymphatic glands. The editors of the *Lancet* observe that it is obvious the condition may follow any extensive obstruction of the inguinal lymphatics, and that the reason it does not more commonly follow removal of lymphatic glands is probably because it is very rare for all the glands to be removed. They point out the difficulties of treatment. Some improvement follows extensive removal of the hypertrophied tissue, but as the obstruction continues there is a great tendency to a re-formation of the condition. Godlee recently, in a case in which there was dilatation of the inguinal lymphatics due to obstruction of the thoracic duct, effected an anastomosis between one of the dilated lymphatics and the saphenous vein, with the result that the dilated lymphatics subsided. In three of zum Busch's cases the enlargement of the prepuce, penis, and scrotum followed extensive operations for suppurating buboes. The fourth affected a woman who had had similar operations and also an erysipelatous affection of the vulva. Riedel has published two similar cases and advises surgeons to avoid too extensive operations for inflamed glands in the groin. The author mentions one case in which the upper extremity was similarly affected after removal of axillary glands by a French surgeon.

**The Treatment of Gonorrhœa.**—C. Leedham-Green [5] has considered this subject. The abortive treatment is not recommended, for the gonococci, even within a few hours of the inoculation, penetrate between the epithelial cells to the deeper parts of the mucous membrane, where they are protected even from the strongest antiseptics that can be applied, and the use of strong antiseptics induces a violent inflammatory reaction which is likely to aggravate the disorder. In acute anterior urethritis local remedies may be used from the first in most cases. There are two contra-indications however to their use, viz., firstly, an exceptionally acute inflammation as evidenced by much œdema of the penis and prepuce, excessive chordee, and blood stained secretion, and secondly, the presence of epididymitis. The patient will have to make the applications himself, and he should be provided with the best form of syringe. The syringe should have a large conical nozzle which will effectually plug the meatus, and must hold enough fluid to completely distend the urethra so that all the folds in the mucous membrane may be obliterated. When not in use it should be kept in carbolic lotion. After micturition the urethra should be two or three times washed out with water to remove all traces of urine, for almost all the injection fluids are readily decomposed by urine. The syringe should hold 10 c.c. of fluid, and a syringeful should be injected into the urethra, unless that amount caused pain from distension. If the fluid is non-irritating, such as a solution of protargol or sulphate of thallin, it should be retained for 10 minutes by compressing the meatus. Astringent fluids should not be retained so long. The author usually starts with injections of $\frac{1}{4}$ per cent. protargol solution three times a day. Usually the discharge rapidly diminishes under it, and the pain and priapism disappear. Then the strength of the solution may be increased, *e.g.*, at the end of the third day, to $\frac{1}{2}$ per cent. solution, at the end of a week to a $\frac{3}{4}$ per cent., and on the tenth day to 1 per cent. This is only to be done if the injection is well borne. If protargol causes pain, then sulphate of thallin solution, in $\frac{1}{2}$ to 1 per cent. strength may be used. Later, when the discharge has become scanty and mucoid, astringent injections may be used. Thus, in the second week, permanganate of potash solution may be used twice a day of 1 in 10,000 strength, and in the evening nitrate of silver, 1 in 10,000. The strength of the latter may be gradually increased. So long as the urine first passed in the morning contains "urethral shreds" the urethra is to be regarded as still inflamed. Occasionally these threads will be found to consist only of epithelial cells and mucin, and such threads may be from a catarrh kept up by the astringent injections. In that case local treatment should be stopped for a time and the effects watched. If during treatment acute posterior urethritis sets in, with hæmaturia, frequent micturition, and seminal emissions, all injections should be stopped. Salicylate of soda, by maintaining acidity of the urine, acts as a prophylactic against cystitis.

**Chronic Urethral Discharge.**—H. M. Christian,[6] in a paper on this subject, points out that it is a popular error to designate as "gleet" all forms of chronic urethral discharge. There are three different forms of the discharge, each depending upon a distinct pathological condition and requiring a distinct line of treatment. True "gleet" is the variety most frequently met with. In this the discharge is scanty, mucoid or muco-purulent, yellowish in colour, staining the linen yellow, and showing under the microscope pus, epithelium, and fibrous shreds. This discharge is the result of gonorrhœa and depends on the presence in the urethra of one or more areas of chronic inflammation, which are superficial in the earlier cases, but in protracted cases spread deeper and cause either "strictures of large calibre," or later true organic stricture. In the posterior urethra the affection causing gleet is chronic follicular prostatitis. The urine in true gleet is clear, but contains clap shreds in abundance which fall to the bottom of the glass. The second form of discharge, quite frequently met with, is that known as prostatorrhœa. Various writers have described the character of this discharge differently. The writer agrees with those who state that the discharge is of normal prostatic fluid, free from pus, and arising from purely functional disorders of the gland. The discharge is milky, and microscopically is found to contain amyloid corpuscles, refractive corpuscles, and amorphous phosphates, and can be obtained by massage of the prostate. The gland is usually congested, and the cause of the discharge is excessive and irregular sexual indulgence. The discharge often appears after straining at stool. The urine does not contain clap threads, but is apt to contain numbers of comma-shaped hook-like shreds which float in the urine. The third form of urethral discharge is that named urethrorrhœa or chronic urethral moisture. The patient complains of persistent moisture about the meatus. The condition is due to an atonic relaxed condition of the urethral mucous membrane, and is a sequel of long-standing gonorrhœa. The discharge is non-contagious, and consists of cylindrical epithelium and mucus. The urine is clear.

**The Treatment of Internal Hæmorrhoids.**—W. Duff-Bullard, New York,[7] describes and criticises the four well-recognised methods of operative interference for internal hæmorrhoids. He states that it is beyond his comprehension why two of these methods, viz., that by injections and the Whitehead operation are ever employed. Every hospital surgeon, he says, must have had opportunity of observing for himself serious life-long injuries to the performance of the normal functions of the rectum, resulting from these forms of treatment. Referring to the Whitehead operation the author points out that it is not a simple matter to dissect off mucous membrane from underlying tissues, especially if engorged and bleeding. A case is mentioned in which incontinence of fæces resulted from removal of the external sphincter in the operation. Some of the sutures are very likely to pull out during coughing, vomiting, or at defœcation, and the result is an ulcer. If all the sutures pull out, as sometimes happens, a stricture can hardly be avoided. An example of this is quoted. The ligature operation and that with the clamp and cautery are the two recommended. Several deaths have resulted from the slipping of ligatures. The cautery operation has some advantages the writer thinks. The pain in the ligature operation is relatively more constant and severe, convalescence more prolonged, and vesical tenesmus more marked.

**Anal Fissure.**—Gussenbauer[8] considers that the best treatment for anal fissure is that by stretching the sphincter. Incision of the fissure, especially if combined with complete division of the sphincter, he regards as a serious operation in which there is considerable hæmorrhage and danger of septic inflammation. Stretching the sphincter cures the spasm and the fissure simultaneously in a few days or a week. After the operation pain is present only for an hour or two. The hæmorrhage is usually slight and most often absent. Defœcation is painless. The fissure heals in from a few days to a fortnight. Continence of fæces is invariably regained in a few days. Deep anæsthesia is required, as even after the corneal reflex has disappeared stretching the sphincter may cause spasm of the glottis. Local anæsthetics are useless. K. Svehla,[9] of Prague, states that in children anal fissure can cause symptoms which may lead to the erroneous diagnosis of coxitis, such as displacement of and pain in the lower extremity and knee, pain in movement of the hip and often on pressing the trochanters and heel, and compensatory scoliosis. Passive mobility is retained, but it is also retained in early hip disease. The explanation of the above symptoms is that by flexion, adduction, and rotation of the hip-joint, the buttocks are separated, and rubbing of the fissure avoided, and by the reflex contraction of muscles due to the pain the limb is fixed in the above position. By striking the heel or trochanters the buttocks are shaken and the fissure thus irritated. As the children get pain every time they sit down, the special pain with defœcation is apt to be overlooked. In one of the five cited cases a plaster of Paris bandage had been applied at a children's hospital owing to a wrong diagnosis of hip disease having been made.

[4] Lancet, March 8, 1902, p. 665. [5] Midland Med. Jour., May 1902, p. 102. [6] Amer. Jour. Med. Sc., March 1902, p. 481. [7] Med. Rec., March 29, 1902, p. 491. [8] Vide Epitome No. 174, B. M. J., Mar. 15, 1902. [9] Vide abst. Centralb. f. Chir., March 15, 1902, p. 309.

